# Relationship between intact HIV-1 proviruses in circulating CD4^+^ T cells and rebound viruses emerging during treatment interruption

**DOI:** 10.1073/pnas.1813512115

**Published:** 2018-11-12

**Authors:** Ching-Lan Lu, Joy A. Pai, Lilian Nogueira, Pilar Mendoza, Henning Gruell, Thiago Y. Oliveira, John Barton, Julio C. C. Lorenzi, Yehuda Z. Cohen, Lillian B. Cohn, Florian Klein, Marina Caskey, Michel C. Nussenzweig, Mila Jankovic

**Affiliations:** ^a^Laboratory of Molecular Immunology, The Rockefeller University, New York, NY 10065;; ^b^Laboratory of Experimental Immunology, Institute of Virology, University Hospital Cologne, 50935 Cologne, Germany;; ^c^Department I of Internal Medicine, University Hospital Cologne, 50931 Cologne, Germany;; ^d^German Center for Infection Research, 50931 Cologne, Germany;; ^e^Department of Physics and Astronomy, University of California, Riverside, CA 92521;; ^f^Center for Molecular Medicine Cologne, University of Cologne, 50931 Cologne, Germany;; ^g^Howard Hughes Medical Institute, The Rockefeller University, New York, NY 10065

**Keywords:** HIV, latent reservoir, sequencing, analytical treatment interruption

## Abstract

Combination antiretroviral therapy controls but does not cure HIV-1 infection because a small fraction of cells harbor latent viruses that can produce rebound viremia when therapy is interrupted. The circulating latent virus reservoir has been documented by a variety of methods, most prominently by viral outgrowth assays (VOAs) in which CD4^+^ T cells are activated to produce virus in vitro, or more recently by amplifying proviral near full-length (NFL) sequences from DNA. Analysis of samples obtained in clinical studies in which individuals underwent analytical treatment interruption (ATI), showed little if any overlap between circulating latent viruses obtained from outgrowth cultures and rebound viruses from plasma. To determine whether intact proviruses amplified from DNA are more closely related to rebound viruses than those obtained from VOAs, we assayed 12 individuals who underwent ATI after infusion of a combination of two monoclonal anti–HIV-1 antibodies. A total of 435 intact proviruses obtained by NFL sequencing were compared with 650 latent viruses from VOAs and 246 plasma rebound viruses. Although, intact NFL and outgrowth culture sequences showed similar levels of stability and diversity with 39% overlap, the size of the reservoir estimated from NFL sequencing was larger than and did not correlate with VOAs. Finally, intact proviruses documented by NFL sequencing showed no sequence overlap with rebound viruses; however, they appear to contribute to recombinant viruses found in plasma during rebound.

## Significance

The HIV-1 latent reservoir is the major barrier to cure. Analysis of the replication competent latent reservoir that can be induced in viral outgrowth assays (VOAs) showed little or no overlap with HIV viruses that emerge in plasma after treatment interruption. To determine whether intact proviruses amplified from DNA are more closely related to rebound viruses than those obtained from VOA, we sequenced HIV proviral genomes from CD4^+^ T cells of individuals who underwent analytical treatment interruption. We find that intact proviruses obtained from DNA overlap in part with those obtained by VOA, but do not overlap with rebound viruses. However, nearly half of all rebound sequences could be accounted for in part by recombination of intact near full-length sequences.

During the HIV-1 infection cycle, viral DNA is inserted in the CD4^+^ T cells as a provirus and is then actively transcribed to produce new virions. In the vast majority of infected cells, this burst of HIV-1 production leads to cell death by apoptosis or pyroptosis (1). A far less frequent alternative fate is for the provirus to be suppressed and to become latent (2). Upon treatment interruption, latent proviruses are stochastically activated leading to rebound viremia and the requirement for life-long therapy for HIV-1 infection (3).

Although numerous methods have been evaluated to measure the latent reservoir, viral outgrowth assays (VOAs) are considered to be the gold standard (2, 4). In this assay, infected donor CD4^+^ T cells are activated in vitro to induce HIV-1 production in the presence of uninfected recipient CD4^+^ T cells. When performed at limiting dilution, and combined with sequence analysis of the emerging viruses, VOAs can yield both quantitative and qualitative information about the latent reservoir (5–7).

When the reservoir is assayed by VOAs, individuals that have achieved viral suppression on antiretroviral therapy (ART) are typically found to harbor 1 between 10^5^–10^6^ circulating CD4^+^ T cells that contain replication competent latent proviruses (8). Longitudinal studies have estimated that the half-life of the latently infected CD4^+^ T cells in the HIV-1 reservoir is 44 mo (9, 10). However, there are several caveats to interpreting the results of the VOAs: (*i*) The assay is highly variable and any difference of less than sixfold is not considered to be significant (10); (*ii*) a single round of stimulation captures only a fraction of the latent cells that can be reactivated (6); (*iii*) individual latent cells differ in their requirements for reactivation (11); (*iv*) VOAs are typically performed on cells derived from blood and this compartment may not be representative of the entire latent reservoir (12); and (*v*) the requirements for reactivation in vitro and in vivo may differ significantly (13, 14).

Sequencing intact viral genomes from limiting dilution CD4^+^ T cell DNA samples is a recently developed alternative method to document the HIV-1 reservoir (11, 15). An advantage of this sequencing method is that it does not require latent virus reactivation in vitro. However, it too is subject to the same sampling caveats as VOAs, and the additional question of whether intact proviruses contribute to rebound viremia during treatment interruption.

A small number of intact proviral sequences obtained by near full-length (NFL) sequencing have been compared with VOAs and rebound viruses in a clinical trial where individuals underwent analytical treatment interruption (ATI) after monotherapy with the 3BNC117 monoclonal anti–HIV-1 antibody (16). To further examine the relationship between proviruses obtained from circulating CD4^+^ T cells by NFL sequencing and the viruses that emerge from the reservoir during treatment interruption, we analyzed samples from 12 individuals enrolled in a clinical trial that involved ATI after infusion of a combination of two broadly neutralizing monoclonal antibodies (bNAbs) (17).

## Results

**Fig. 1.**
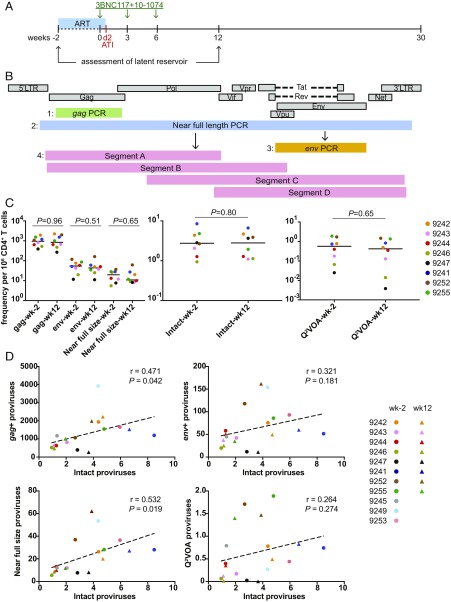
Quantitative analysis of the latent reservoir during treatment interruption. (*A*) Study design. Green arrows indicate combination bNAb infusion. Black arrows indicate the time points that were sampled. (*B*) NFL HIV-1 genome sequencing strategy (11). All viruses that had deletion in *env* were excluded from further analysis. (*C*) Comparison of reservoir measurements. Graph shows frequency per million CD4^+^ T cells at the preinfusion (wk-2) and week-12 (wk12) time points: *gag*^+^ proviruses (gag), *env*^+^ proviruses (env), near full-size proviruses (near full size) (*Left*), intact proviruses (intact) (*Middle*), and inducible proviruses (Q^2^VOA) (17) (*Right*). Each dot represents a different participant. Horizontal bars indicate median values. Statistical significance was determined using two-tailed Mann–Whitney *U* test. (*D*) Pearson correlation between frequency of intact proviruses and other reservoir measurements at the preinfusion (wk-2) (circles) and wk12 (triangles) time points. Participant 9254 was excluded from the quantitative analysis because of inadequate sample availability.

3BNC117 and 10-1074 are broad and potent monoclonal anti–HIV-1 antibodies that target independent nonoverlapping sites on the HIV-1 envelope spike (18, 19). The combination of the two antibodies was administered to 15 individuals undergoing ATI, 2 d before, and 3 and 6 wk after ATI (Fig. 1*A*) (17). Leukapheresis was performed 2 wk before and 12 wk after ATI, and plasma was obtained for single genome analysis (SGA) at the time of rebound.

NFL proviral genomes were amplified from DNA extracted from purified CD4^+^ T cells obtained from the two leukapheresis samples from nine individuals that maintained viral suppression for >12 wk after ATI, including two that remained virologically suppressed during the entire observation period (>30 wk, participants 9254 and 9255) (11, 17, 20). A single preinfusion sample was also available for three additional individuals that experienced viral rebound within 12 wk of ATI (participants 9245, 9249, and 9253) (17). Qualitative and quantitative viral outgrowth assay (Q^2^VOA) was performed on all of these samples (17). Participant 9254 was excluded from the quantitative part of the analysis because of inadequate sample availability.

HIV-1 DNA content was initially measured by performing limiting dilution *gag* PCR (Fig. 1*B*) (11). NFL genomes were amplified from DNA aliquots containing a single HIV-1 genome (Fig. 1*B*). To increase efficiency, only samples containing full-length *env* fragment were further processed and subjected to sequence analysis (Fig. 1*B*).

Overall, the amount of HIV-1 DNA in CD4^+^ T cells in circulation did not change significantly during the observation period. On average *gag*^+^ proviruses were found at a frequency of 942 and 841 out of 10^6^ CD4^+^ T cells at the preinfusion and week-12 time points, respectively (Fig. 1*C* and *SI Appendix*, Table S1). Although individual participants showed some variation in the frequency of *gag*^+^ proviruses at the two time points, the degree of variation was consistent with 2.5-fold variability in the assay (Fig. 1*C* and *SI Appendix*, Table S1).

Defective HIV-1 proviruses frequently carry deletions in *env* (11). We found that the median number of proviruses containing full-length *env* was 21 times lower than *gag*^*+*^ proviruses for both preinfusion and week-12 time points (Fig. 1*C* and *SI Appendix*, Table S1). Proviruses with a near full-size HIV-1 genome were found at a still lower frequency of 20 and 11 out of 10^6^ CD4^+^ T cells at the preinfusion and week-12 time points, respectively (Fig. 1*C* and *SI Appendix*, Table S1). The difference between the number of CD4^+^ T cells containing *env* and a near full-size HIV-1 genome is expected because a fraction of defective proviruses retains intact *env* (21). Similar to *gag* or *env*, there was no significant difference in the frequency of cells containing near full-size HIV genomes between the two time points (Fig. 1*C* and *SI Appendix*, Table S1).

To determine the frequency of intact proviruses in circulating CD4^+^ T cells, we sequenced all near full-size HIV-1 proviruses and counted only those with intact reading frames, packaging signals, and major splice donors (MSDs). Intact proviruses were found at a median frequency of 2.8 out of 10^6^ CD4^+^ T cells at both time points, which is 5.9- and 6.9-fold lower than the number of proviruses containing near full-size HIV-1 genomes at the preinfusion and 12-wk time points, respectively (Fig. 1*C* and *SI Appendix*, Table S1). Overall, on average only 1 out of 336 or 300 *gag*^+^ proviruses were intact at the preinfusion and week-12 time points. Nevertheless, there was a positive correlation between the number of intact and *gag*^+^ proviruses, and also between intact and near full-size proviruses (*P* = 0.042, *r* = 0.471 and *P* = 0.019, *r* = 0.532, respectively, Fig. 1*D*).

**Fig. 2.**
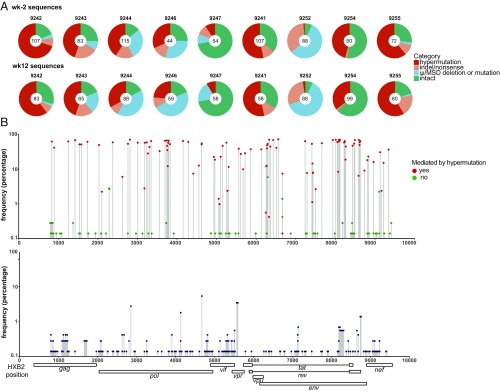
Sequence analysis of near full-size proviruses. (*A*) Pie charts summarize sequence analysis at the preinfusion (wk-2) and week 12 (wk12) time points. The number in the middle of the pie represents the number of near full-size proviruses sequenced. Pie slices depict the proportion of sequences for each participant that were intact or had different lethal defects, including premature stop codons mediated by hypermutation, single nucleotide indels, nonsense mutations, packaging signal (ψ) deletions, and MSD site mutations. (*B*) Scatterplot showing the frequency and location of stop codons mediated by hypermutation (red circles) or by other mechanisms (green circles) (*Upper*) and indels (blue squares) (*Lower*). The location of stop codons and indels was determined using HXB2 genome as reference. Frequency is expressed as percentage of all defective sequences.

The difference between the number of proviruses with near full-sized genomes and intact sequences can be explained by a combination of hypermutation, small insertions and deletions (indels), and defects in the packaging site and/or major splice donor. The relative contribution of each of these differed significantly between individuals but was similar at the two time points for each participant. For example, packaging site and major splice site defects were dominant in participant 9252, but these defects were not found in participant 9241 (Fig. 2*A* and *SI Appendix*, Table S2). Overall, hypermutation accounted for ∼55% of all defective near full-sized genomes, but this too varied between individuals. After hypermutation, the next most frequent source of defective proviruses was packaging site and/or major splice donor mutation or deletion at an overall rate of ∼27%, with the remainder accounted for by indel/nonsense mutations (*SI Appendix*, Table S2).

Although there were hotspots for mutations that produced premature stop codons, most of which correspond to APOBEC3 target sites, hypermutations were found throughout the HIV-1 genome (Fig. 2*B*, *Upper*). On average, each individual hypermutated sequence carried 54 in-frame stop codon mutations. In contrast, it was unusual to find multiple indels in a single sequence. In addition, the location of the indels did not appear to correspond to the APOBEC3 mutation hotspots (Fig. 2*B*, *Lower*).

Compared with NFL sequencing, Q^2^VOA measurements performed on the same samples showed fewer infectious units per million (*P* < 0.001) (*SI Appendix*, Fig. S1 and Table S1). The median number of inducible viruses enumerated by Q^2^VOA was 8.5- and 8.4-fold lower than the NFL measurement at the preinfusion and week-12 time points, respectively (Fig. 1*C* and *SI Appendix*, Table S1). As noted by others (21), the difference between the two measurements in individual participants varied and there was no direct correlation between the two measurements (Fig. 1*D* and *SI Appendix*, Table S1). For example, when both time points are taken together for participant 9247, 0.87% of *gag*^+^ proviruses contain an intact provirus, but only 0.004% of *gag*^+^ proviruses have a virus that emerges in the Q^2^VOA (*SI Appendix*, Fig. S2). In contrast, in participant 9255, the percentage of *gag*^+^ proviruses that contain intact provirus is only two times higher than the percentage of viruses that emerges in the Q^2^VOA (0.26% and 0.13%, respectively) (*SI Appendix*, Fig. S2). Although the size of the reservoir estimated by the two methods was different, the overall diversity of HIV-1 sequences was similar (*P* = 0.79) (*SI Appendix*, Fig. S3). Neither the number of intact proviruses at the preinfusion time point nor the change in the number of intact proviruses between the two time points correlated with time to rebound (*P* = 0.69 and 0.07, respectively).

**Fig. 3.**
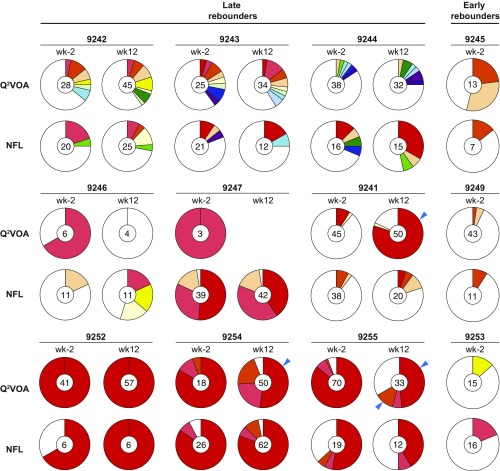
Qualitative analysis of the circulating latent reservoir. Pie charts show the clonal distribution of *env* sequences derived from Q^2^VOA (17) or NFL sequencing for each participant at the preinfusion (wk-2) and week 12 (wk12) time points. The number in the middle indicates the total number of *env* sequences analyzed. White slices represent unique sequences isolated only once across both time points from both Q^2^VOA and NFL sequencing (singles), and colored slices represent identical sequences that appear more than once (clones). The colors of the slices represent identical sequences found in Q^2^VOA and in NFL. Blue arrows indicate clones that show significant differences between the time points (17).

**Fig. 4.**
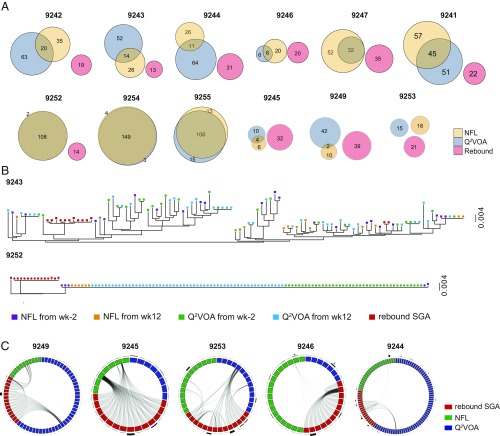
Comparisons of the circulating latent reservoir and rebound viruses. (*A*) Diagrams show the overlap between *env* sequences obtained from Q^2^VOA (blue), NFL sequencing (yellow), and rebound plasma SGA or PBMC outgrowth culture (red) (17). The intersection of the NFL and Q^2^VOA circles represents the number of identical *env* sequences belonging to clones obtained by both methods. Sequences obtained from the preinfusion and week 12 were combined. (*B*) Maximum likelihood phylogenetic trees of *env* sequences obtained from Q^2^VOA at preinfusion (green) and week 12 (blue), NFL at the preinfusion (purple) and week 12 (orange), and rebound viruses from SGA or outgrowth cultures (red) from two representative participants. Additional participants are shown in *SI Appendix*, Fig. S4. (*C*) Circos plots depicting recombination events between *env* sequences derived from Q^2^VOA at preinfusion (blue), intact NFL at preinfusion (green), and rebound plasma SGA (red). Gray lines show the contribution of parent sequences to recombinant sequences. Clonal *env* sequences were collapsed and represented as one virus. The thickness of the black outer bars represents the number of sequences obtained from that particular clone. Asterisks indicate the same *env* sequences between intact NFL and Q^2^VOA sequences.

Q^2^VOA analysis revealed that 56% of the viruses found at the preinfusion and week-12 time points belonged to expanded clones (17). A comparable number of clonal viruses was also found among the intact NFL sequences (53%, *SI Appendix*, Table S3). Overall, 39% of the all intact NFL sequences were identical to Q^2^VOA sequences (Figs. 3 and 4 *A* and *B* and *SI Appendix*, Fig. S4). Similar to Q^2^VOA, identical expanded intact NFL clones appeared at both time points in nearly all of the individuals tested, but the relative representation of each of the clones varied. Large expanded clones that dominated in Q^2^VOA in participants 9252, 9254, and 9255 were also dominant in intact NFL sequences in those individuals. In contrast, there was far less overlap between the two assays in individuals like 9242, 9243, and 9244 that have a more diverse latent reservoir (Figs. 3 and 4 *A* and *B* and *SI Appendix*, Fig. S4). Consistent with these observations there was a strong positive correlation between the percentage of *env* clonality and overlap between Q^2^VOA and intact NFL sequences (*P* = 0.0015, *r* = 0.808, *SI Appendix*, Fig. S5).

When assayed by VOA, the relative distribution of clones is dynamic in that the number of cells that reactivate a specific latent provirus frequently differs between time points (17, 22). For example, individuals 9241, 9254, and 9255 show significant changes in clonal distribution by Q^2^VOA. In contrast, NFL sequencing failed to reveal significant changes in any of the individuals assayed (Fig. 3). The disparity between the two assays is likely due in part to the requirement for reactivation in VOAs and difference in the number of CD4^+^ T cells assayed by the two methods (average 24-fold higher for Q^2^VOA, *SI Appendix*, Fig. S6).

To examine the relationship between circulating intact proviruses documented by NFL sequencing and plasma rebound viruses, we compared *env* sequences obtained from 10 individuals that underwent ATI after infusion of a combination of broadly neutralizing antibodies. The selected individuals included the seven that had two leukapheresis and rebounded late (>12 wk after ATI), and three that rebounded early due to preexisting antibody resistance (17). Although all of the rebound viruses were >96% identical to at least one sequence from the reservoir, we did not find a single instance of 100% *env* identity among 435 intact NFL sequences and 246 rebound viruses obtained by SGA (Fig. 4 *A* and *B* and *SI Appendix*, Fig. S4).

To determine whether rebound sequences could have evolved by accumulating mutations during ATI, we used a mathematical model to simulate this process (16). We found that only 12 out of 246 rebound sequences (*SI Appendix*, Fig. S7, blue bars) could be accounted for by mutation of reservoir sequences (*SI Appendix*, Fig. S7, gray bars). Consistent with a previous report, the observed distance between latent and rebound viruses decreased in 205 out of 246 rebound sequences when the possibility of recombination was included (*SI Appendix*, Fig. S7, yellow bars). Using the 3SEQ recombination algorithm (mol.ax/software/3seq/), we found that 48% of the rebound viruses could be recombinants between intact NFL and/or Q^2^VOA proviruses. For example, in participants 9249, 9253, 9244, and 9246, the “parent” *env* sequences were either from intact NFL or Q^2^VOA, and in participant 9245, the parent sequences were uniquely from intact NFL (Fig. 4*C*). In addition, rebound viral sequences also served as parents for recombination (Fig. 4*C*). There was no discernible pattern to the recombination events. However, among the 12 latent parent sequences, there was only one instance in which the parent virus was part of an expanded clone. Finally, the sensitivity to bNAbs was comparable between recombinants and other rebound viruses. In conclusion, some of the rebound viruses that emerge during ATI appear to be recombinants derived from the circulating latent reservoir characterized by Q^2^VOA or NFL sequencing.

## Discussion

Long-lived integrated proviruses represent the key barrier to HIV-1 cure (2). Several different assays have been used to try to characterize and measure the latent reservoir, most prominently nucleic acid based and viral outgrowth assays (23). Although these assay have produced a great deal of information on latent proviruses found in circulating CD4^+^ T cells, the precise relationship between these viruses and those that emerge in HIV-1–infected individuals during ART interruption has not been defined. To add to the dataset that addresses this important question, we have compared 435 intact NFL proviruses obtained from circulating CD4^+^ T cells by NFL sequencing to 650 Q^2^VOA and 246 plasma rebound viruses from individuals enrolled in a clinical trial of combination immunotherapy with broadly neutralizing antibodies 3BNC117 and 10-1074 (17).

Overall Q^2^VOA and NFL sequencing yield overlapping and similarly diverse sets of viruses. As might be expected, the overlap between the sequences obtained by the two methods was most significant in individuals with a more clonal and less diverse reservoir. One of the differences between the two methods was that clones obtained from NFL sequencing appeared to be more stable between the two time points. For example, participants 9241, 9254, and 9255 showed significant clonal variation by Q^2^VOA not found by NFL sequencing. These qualitative differences could be due to stochastic activation of latent viruses in vitro and/or to sampling error because the total number of cells sampled in the NFL sequencing was on average 24-fold lower than in Q^2^VOA.

There were also significant quantitative differences between proviruses enumerated by NFL sequencing and Q^2^VOA. In the individuals assayed, Q^2^VOA underestimates the size of the latent reservoir by an average of 8.5-fold, but there is tremendous variation between individuals ranging from 1.4- to 915-fold. This finding is consistent with previous reports showing that VOA underestimates the size of the latent reservoir, and that there is no clear correlation between the two methods (6, 11, 21). These quantitative differences could be accounted for if some of the intact proviruses documented by NFL sequencing cannot be reactivated or are simply defective in some way that has not been detected. However, in all instances reported to date, proviruses reconstructed based on intact NFL sequences were productively infectious in vitro (11). An alternative and nonexclusive explanation for the disparity between the measurements is that the requirements for latent virus reactivation are both cell or stimulus specific such that only a seemingly random fraction of all latent viruses are reactivated by a particular stimulus in vitro (6).

The vast majority of near full-sized genomes we sequenced, 83% were defective due to hypermutation or mutant packaging signals or major splice donors. Defective proviruses can produce protein products that are targeted by cytotoxic T cells (24); however, we found little fluctuation in the number of these proviruses or the types of defects they carry between two time points.

Three recent clinical studies that included ATI after bNAb infusion evaluated the relationship between circulating latent viruses documented by VOA and rebound viruses (16, 17, 25). A total of 1,411 VOA and 682 rebound viruses were characterized from 30 individuals undergoing ATI. A small number of intact NFL sequences were also obtained by Cohen et al. (16). In all, there were only 13 instances of overlap between latent and rebound viruses and this occurred in only 4 of the 30 individuals. Although there are antibody-dependent selective changes in the rebound viruses, viral sensitivity to the bNAb and time to rebound did not influence the results (16). In addition, Cohen et al. found that the differences between the latent and rebound viruses could not be accounted for by HIV-1 mutation, but instead, it appeared that rebound viruses were frequently composed of recombinants between viruses emerging in Q^2^VOA (16).

Our results comparing 435 *env* sequences from intact proviruses with 246 plasma-derived rebound *envs* add to this growing body of data. Although proviruses found in circulation can contribute to rebound (26), we did not observe direct overlap between intact NFL and rebound sequences. Instead, 48% of all rebound *env* sequences in 10 individuals could be accounted for in part by recombination when both intact NFL and Q^2^VOA sequences are taken into account. This is likely to be an underestimate since we are only considering recombination within *env*. Thus, intact proviral NFL sequences found in the circulating reservoir may be able to contribute to rebound by recombination. Whether similar effects will also be seen in individuals undergoing ATI in the absence of bNAb therapy remains to be determined.

Recombination is a frequent occurrence during active infection (27, 28). We are unable to determine directly whether the recombinant viruses observed during ATI preexist in the reservoir or emerge during rebound. If they do preexist then they must be very rare in circulation (<1%) and may be resident in tissues such as the gut-associated lymphoid tissue (29). Cells carrying these proviruses could be activated to produce them by stimuli that are either unavailable to, or simply fail to activate cells in the blood. For example, tissue resident CD4^+^ T cells in the gut are exposed to far higher concentrations of bacterial products than cells in circulation. However, VOAs and/or NFL sequencing have not been performed on tissues from individuals undergoing ATI and so this possibility remains speculative.

The alternative equally speculative idea is that latent proviruses are generally unable to propagate robust infection in vivo, and that establishment of proviral latency is facilitated by lower overall viral fitness. According to this hypothesis, recombination during rebound would facilitate selection of HIV-1 variants capable of producing robust viremia in vivo. The availability of large numbers of latent and rebound HIV-1 viruses should enable the testing of this idea.

In conclusion, Q^2^VOA and NFL sequencing assays provide complementary sets of information on HIV-1 proviruses in the latent reservoir. However, the significance of this information in assessing therapies aimed at HIV-1 cure is unclear and requires further investigation (26). The results emphasize the importance of ATI in evaluating therapies aimed at long-term remission or cure of HIV-1 infection.

## Materials and Methods

### Study Subjects.

Study participants were enrolled in an open-label phase 1b study in which the combination of two anti–HIV-1 bNAbs were administered during ATI (17). The protocol was approved by the Food and Drug Administration, the Paul Ehrlich Institute in Germany, and the Institutional Review Boards at The Rockefeller University and the University of Cologne. All participants provided written informed consent before participation in the study and the study was conducted in accordance with Good Clinical Practice.

### Q^2^VOA.

The Q^2^VOA was performed as previously described (5, 17).

### Rebound Outgrowth Cultures.

The rebound outgrowth cultures were performed as previous described (17).

### DNA Extraction and NFL HIV-1 Genome Sequencing.

Peripheral blood mononuclear cells (PBMCs) were obtained by leukapheresis at the preinfusion and week 12. DNA was extracted from 1 to 10 × 10^6^ CD4^+^ T cells using Qiagen Gentra Purgene Cell Kit. Near full-length HIV-1 genome was generated as previously described (11, 16, 20). Briefly, DNA was subjected to a limiting-dilution *gag* PCR using 5′GagIn; 5′-GGGAAAAAATTCGGTTAAGGCC-3′ and 3′GagIn 5′-CGAGGGGTCGTTGCCAAAGA-3′ in the first round and seminested primer 3′GagInIn 5′-GGGGCTGTTGGCTCTGGT-3′ in the second round with Platinum Taq polymerase (Invitrogen). Seminested *gag* PCR conditions were 94 °C for 2 min; 50 cycles of 94 °C for 30 s, 61 °C for 30 s, and 68 °C for 3 min; and 68 °C for 10 min. PCR products were visualized and quantified using 1% agarose gels. DNA dilutions wherein <30% of the *gag* PCR wells were positive, were selected for further analysis because they have more than an 80% probability of containing a single copy of HIV DNA in each PCR based on the Poisson distribution. NFL outer PCR was performed on DNA diluted to single genome levels using Platinum Taq High Fidelity polymerase, and 1-μL aliquots were subjected to nested *env* PCR using envB5out 5′-TAGAGCCCTGGAAGCATCCAGGAAG-3′ and envB3out 5′-TTGCTACTTGTGATTGCTCCATGT-3′ (5). NFL samples containing ∼3,000-bp amplicons were subjected to four-segment PCR (A–D) (11) and then visualized on 0.7% agarose gels. If the size of either segment A + C, A + D, B + C, or B + D was correct, two-segment PCR products were combined and then subjected to library preparation and sequencing (5). Paired-end reads were adapter trimmed and filtered for quality with a Phred score cutoff of 10, using trimgalore v0.4.1 and cutadapt v1.11. De novo assembly of the reads into scaffolds was performed using SPAdes genome assembler v3.9.0. Scaffolds were then used to find the closest HIV reference by BLAST, either against a local database of HIV *env* annotations in the case of *env* assembly, or the National Center for Biotechnology Information database in the case of full genome assembly. The closest reference and the scaffolds are then assembled using Mira v4.0.2 to generate an edited reference that more closely reflects the actual sampled sequence. Finally, this edited reference is used as a backbone to assemble the reads.

### Identification of Intact Proviruses.

Assembled sequences were aligned to the HXB2 genome to identify premature stop codons, out-of-frame insertions or deletions (indels), or packaging signal (Ψ) deletions and mutations using custom Python scripts. Sequences containing productive genes and the MSD site are classified as intact, while sequences with a mutated or deleted MSD site are classified as Ψ-MSD deletion/mutation (*SI Appendix*, Fig. S8). Presence of APOBEC-induced G–A hypermutation was determined in the remaining intact NFL proviruses using the Los Alamos HIV Sequence Database Hypermut tool. Sequences not classified as hypermutated are considered defective due to indels/nonsense mutations (*SI Appendix*, Fig. S8). The frequency of intact proviruses was calculated as the number of intact proviruses divided by the total number of CD4^+^ T cells assayed.

### Comparisons of Different Reservoir Measurements.

Fold change between different reservoir measurements was based on the median fold change in individual patients.

### Construction of Phylogenetic Tree.

Nucleotide alignments of intact *env* sequences were translation aligned using ClustalW v2.1 under the BLOSUM cost matrix. Maximum likelihood phylogenetic trees were then generated from these alignments with PhyML v3.1 using the general time-reversible (GTR) model with 1,000 bootstraps. For the combined analysis of sequences from all participants, *env* sequences were aligned using MAFFT v7.309 and clustered using RAxML v8.2.9 under the GTRGAMMA model with 1,000 bootstraps. To analyze changes between reservoir and rebound viruses, *env* sequences were aligned at the amino acid level to a HXB2 reference using ClustalW v2.1.

### Recombination Analysis of *env* Sequences.

Multiple alignment of nucleotide sequences guided by amino acid translations of *env* sequences was performed by TranslatorX (translatorx.co.uk/). *env* sequences from Q^2^VOA, NFL, and rebound sequences were analyzed for the presence of recombination using the 3SEQ recombination algorithm (mol.ax/software/3seq/). Sequences presenting statistical evidence of recombination (rejection of the null hypothesis of clonal evolution) in which parent sequences were derived from the latent reservoir and the “child” sequence was a rebound sequence are represented in a circos plot (circos.ca/).

### Simulation of Mutation Accumulation During Rebound.

The differences between rebound and NFL/Q^2^VOA sequences were analyzed using the stochastic mutation simulation model as previously described (16). The expected number of mutations accumulated during rebound was calculated taking into consideration the time to rebound.

### Statistical Analyses.

Statistical analyses were performed using GraphPad Prism 7.0a for Mac OS X.

## Supplementary Material

Supplementary File
