# Supplementary material for: Relationship between intact HIV-1 proviruses in circulating CD4+ T cells and rebound viruses emerging during treatment interruption
Source: Proc Natl Acad Sci U S A. 2018 Nov 12;115(48):E11341–8. doi: 10.1073/pnas.1813512115 (PMC6275529; doi:10.1073/pnas.1813512115)
Supplement: Supplementary File [file pnas.1813512115.sapp.pdf]

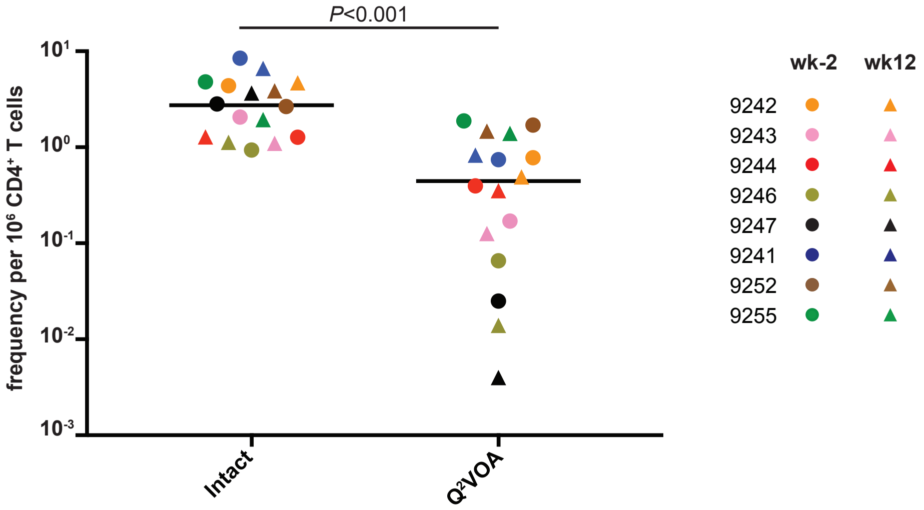

Fig. S1. Comparisons of frequencies of intact proviruses and inducible proviruses.
Scatter plot showing the frequencies of intact proviruses and inducible proviruses measured by NFL sequencing and Q2VOA, respectively.
Each dot represents a different participant. Horizontal bars indicate median values.
Statistical significance was determined using two-tailed Mann-Whitney U test.

www.pnas.org/cgi/doi/10.1073/pnas.1813512115

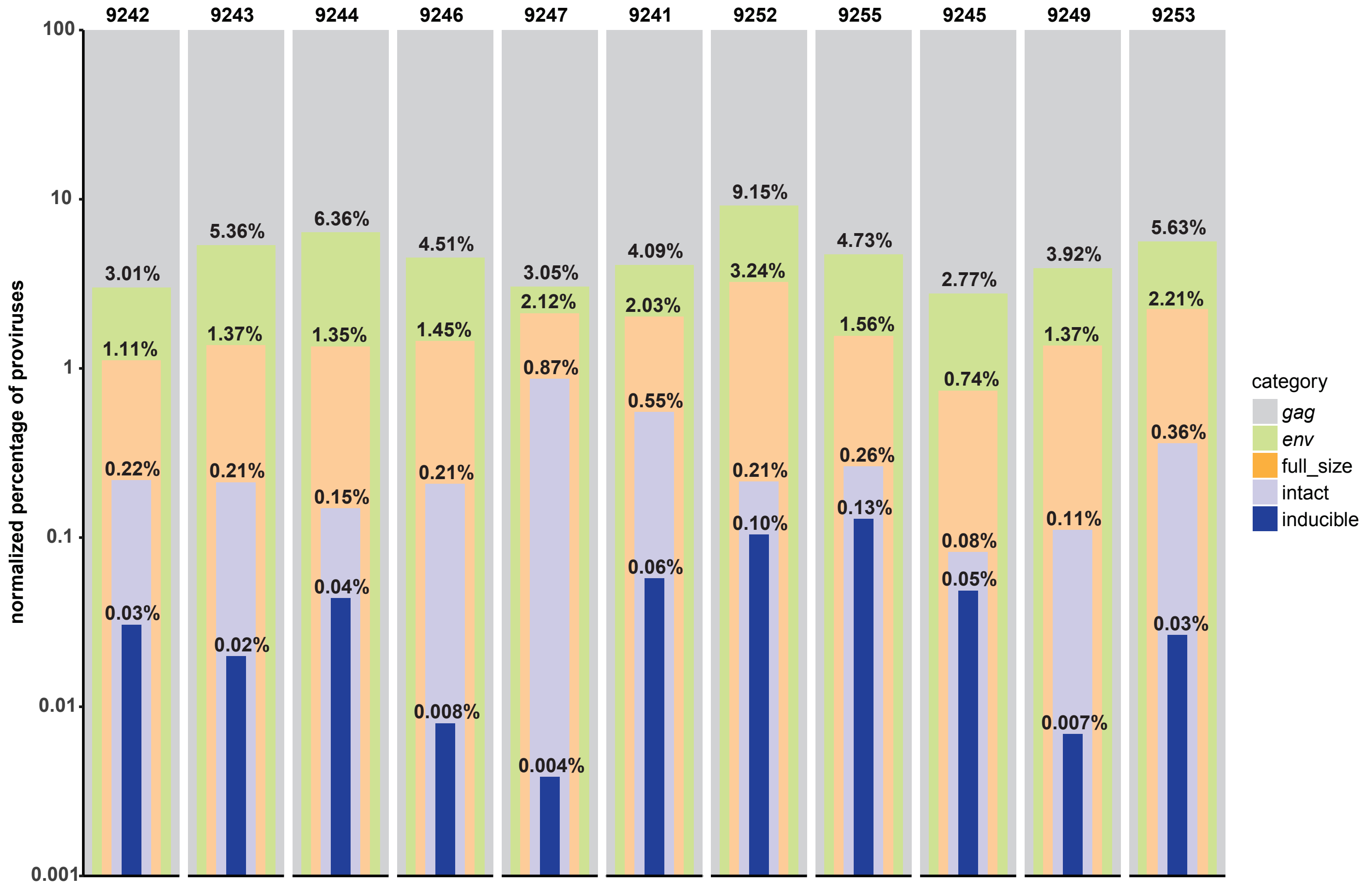

Fig. S2. Comparisons of different reservoir measurements.
Bar graph showing the proportion of env+ proviruses, near full size proviruses, intact proviruses, and inducible proviruses within gag+ proviruses population.

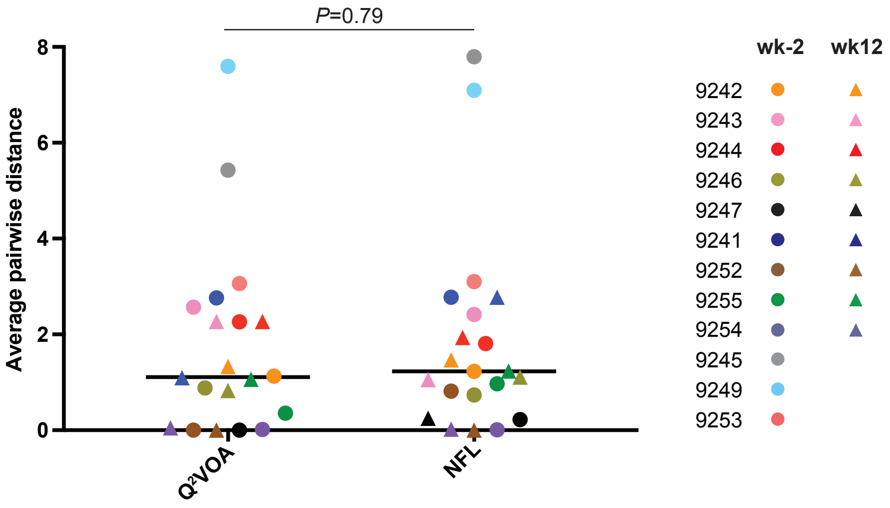

Fig. S3. HIV-1 env sequences diversity.
Scatter plot showing HIV-1 env sequence diversity from Q2VOA or NFL sequencing measured by average pairwise nucleotide distance.
Each dot represents a different participant. Horizontal bars indicate median values.
Statistical significance was determined using two-tailed Mann-Whitney U test.

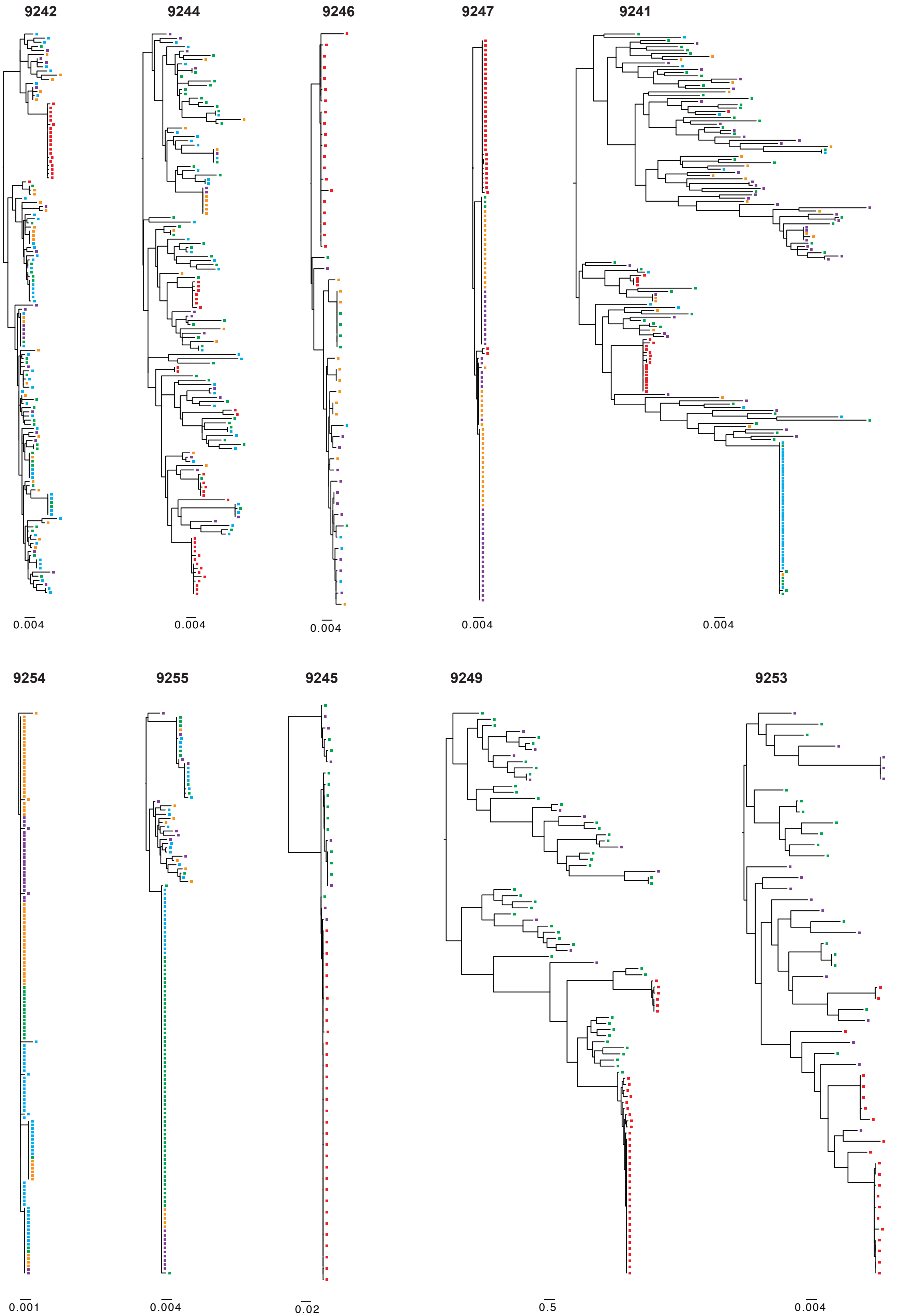

Fig. S4. Phylogenetic trees of env sequences.
Maximum-likelihood phylogenetic trees of env sequences obtained from Q2VOA at the pre-infusion (green) and week 12 (blue), NFL at the pre-infusion (purple) and week 12 (orange), and rebound viruses from SGA or outgrowth cultures (red).

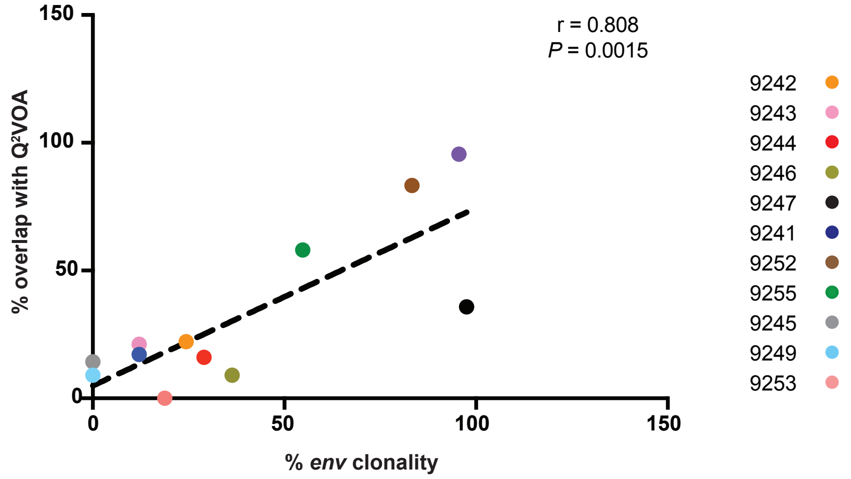

Fig. S5. Correlation between shared sequences from two methods and clonality.
Scatter plot showing Pearson correlation between the percentage of clones and percentage of overlap between Q2VOA and NFL sequencing. Each dot represents a different participant.

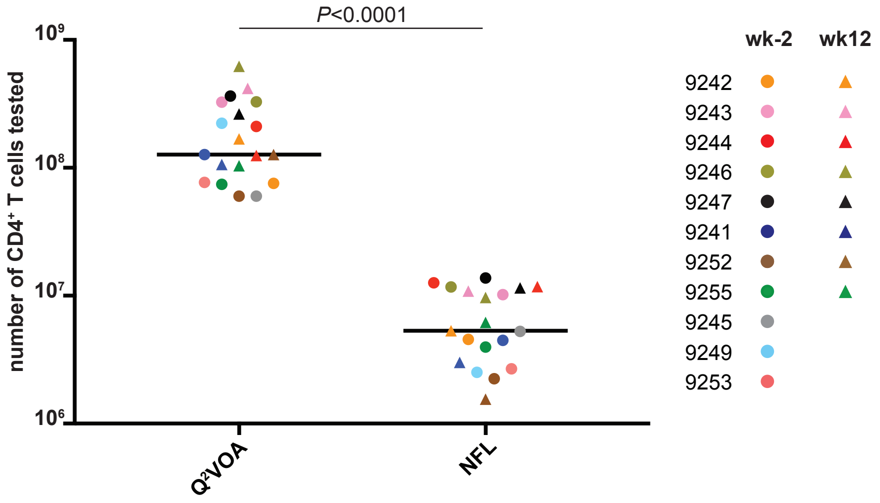

Fig. S6. Number of CD4+ T cells tested.
Scatter plot showing the number of CD4+ T cells tested in Q2VOA and NFL sequencing. Each dot represents a different participant. Horizontal bars indicate median values. Statistical significance was determined using two-tailed Mann-Whitney U test.

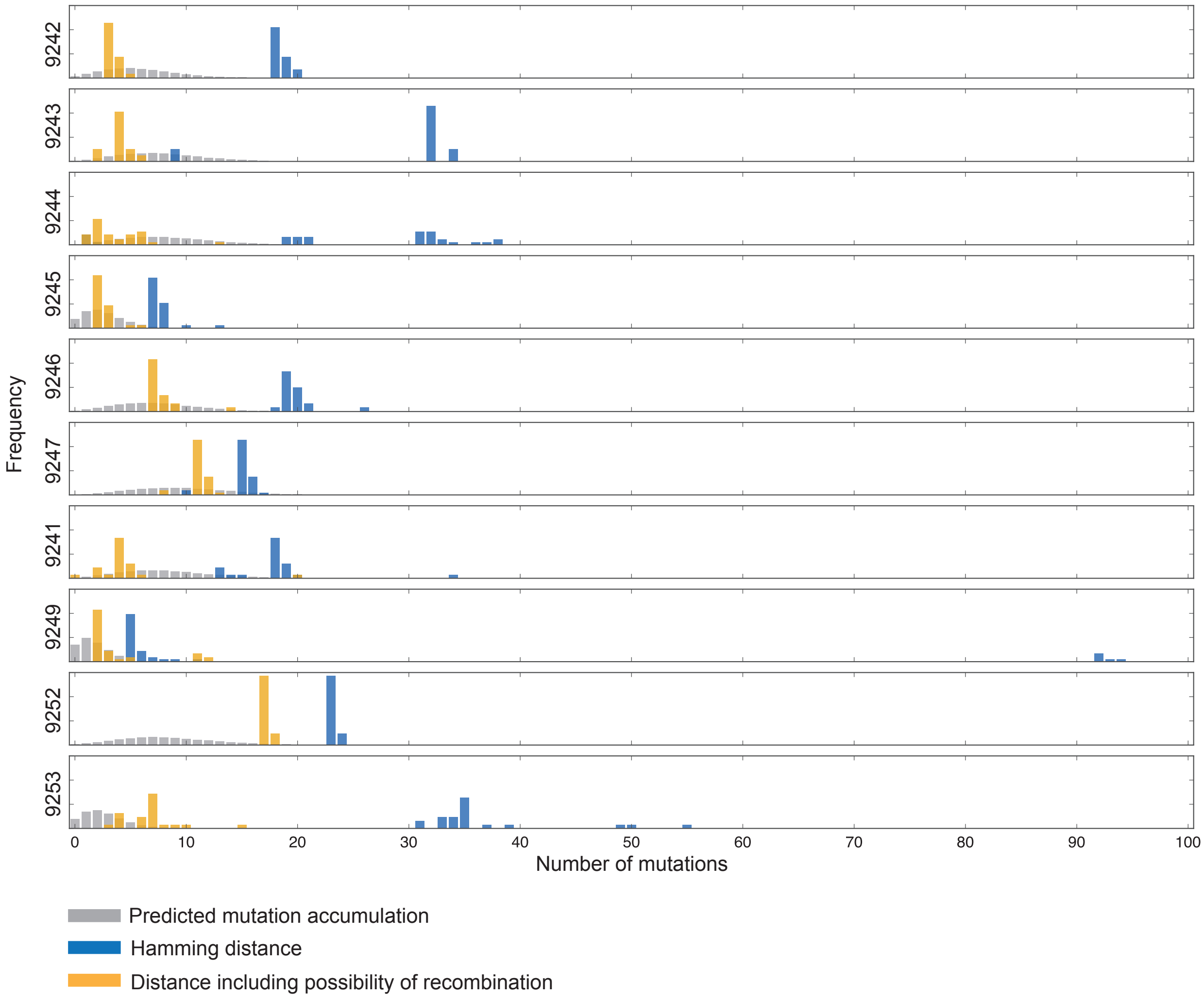

Fig. S7. Relationship between latent and rebound sequences.
Histograms show the proportion of env sequences (y-axis) and number of mutations (x-axis).
The blue bars represent the Hamming distance between rebound and NFL/Q2VOA sequences.
The grey bars represent the predicted distance between rebound and NFL/Q2VOA sequences based on a simulation of mutation accumulation during the ATI period for each participant.
Yellow bars represent the predicted distance between the rebound and NFL/ Q2VOA sequences when the possibility of recombination is included.

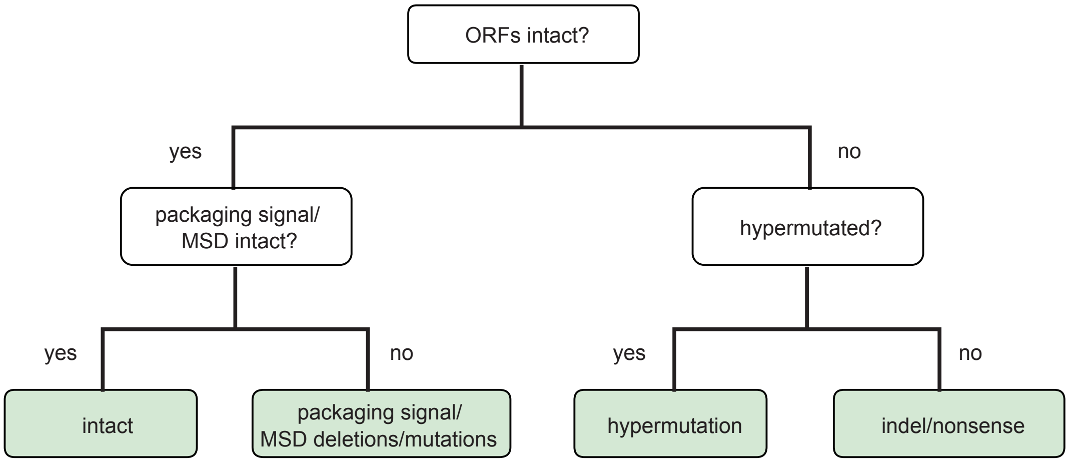

Fig. S8. Process used to identify intact and defective proviruses.
Assembled sequences were aligned to the HXB2 genome to identify premature stop codons, out-of-frame insertions or deletions (indel), or packaging signal (Ψ) and the major splice donor (MSD) site deletions and mutations.

Table 1. Quantitative analysis of the latent reservoir during treatment interruption.

	ID	Weeks to viral rebound	CD4+ T cells tested in Q^2VOA		CD4+ T cells tested in NFL		Gag+ proviruses per 10^6 cells		Env+ proviruses per 10^6 cells		full size proviruses per 10^6 cells		intact proviruses per 10^6 cells		Inducible proviruses per 10^6 cells	
			week -2	week 12	week -2	week 12	week -2	week 12	week -2	week 12	week -2	week 12	week -2	week 12	week -2	week 12
**late rebounder**	**9242**	15	7.56E+07	1.68E+08	4.57E+06	5.32E+06	1938	2229	75.77	49.63	26.28	20.11	4.38	4.70	0.781	0.493
	**9243**	20	3.26E+08	4.18E+08	1.02E+07	1.09E+07	816	674	42.31	37.58	11.98	8.45	2.06	1.10	0.170	0.126
	**9244**	21	2.11E+08	1.25E+08	1.26E+07	1.18E+07	636	646	58.31	50.60	13.09	10.03	1.27	1.28	0.397	0.354
	**9246**	19	3.28E+08	6.22E+08	1.17E+07	9.70E+06	526	475	19.87	25.26	5.57	8.97	0.94	1.13	0.066	0.014
	**9247**	26	3.64E+08	2.64E+08	1.38E+07	1.15E+07	392	268	11.67	11.15	7.68	8.19	2.83	3.66	0.025	0.004
	**9241**	21	1.27E+08	1.06E+08	4.48E+06	3.01E+06	1199	1531	51.52	60.15	28.10	27.25	8.48	6.65	0.743	0.828
	**9252**	22	6.00E+07	1.27E+08	2.25E+06	1.55E+06	1068	1988	117.97	161.79	36.95	62.13	2.67	3.88	1.709	1.470
	**9255**	>30	7.44E+07	1.04E+08	3.97E+06	6.19E+06	1548	1007	85.92	34.91	28.22	11.80	4.79	1.94	1.890	1.400
**early rebounder**	**9245**	5	6.00E+07		5.25E+06		1171		44.98		12.01		1.33		0.79	
	**9249**	3	2.23E+08		2.52E+06		3922		153.84		53.53		4.36		0.27	
	**9253**	5	7.68E+07		2.68E+06		1653		93.11		36.50		5.96		0.44	

Table 2. Analysis of near full size proviruses.

ID	intact proviruses				ψ/MSD mutation or deletion				hypermutation				indel/nonsense				total sequences	
	week -2		week 12		week -2		week 12		week -2		week 12		week -2		week 12		week -2	week 12
	no.	%	no.	%	no.	%	no.	%	no.	%	no.	%	no.	%	no.	%	no.	no.
**9242**	20	18.7	25	30.1	4	3.7	2	2.4	75	70.1	50	60.2	8	7.5	6	7.2	107	83
**9243**	21	25.3	12	18.5	7	8.4	13	20	37	44.6	29	44.6	18	21.7	11	16.9	83	65
**9244**	16	13.9	15	17	33	28.7	36	40.9	47	40.9	29	33	19	16.5	8	9.1	115	88
**9246**	11	25	11	18.6	13	29.5	28	47.5	14	31.8	14	23.7	6	13.6	6	10.2	44	59
**9247**	39	7.2.2	42	72.4	5	9.3	11	19	5	9.3	5	8.6	5	9.3	0	0	54	58
**9241**	38	35.5	20	34.5	0	0	0	0	57	53.3	31	53.4	12	11.2	7	12.1	107	58
**9252**	6	6.8	6	6.8	50	56.8	55	62.5	1	1.1	2	2.3	31	35.2	25	28.4	88	88
**9254**	26	52	62	62.6	0	0	0	0	23	46	35	35.4	1	2	2	2	50	99
**9255**	19	26.4	12	20	2	2.8	1	1.7	44	61.1	35	58.3	7	9.7	12	20	72	60

Table 3. Qualitative analysis of the circulating latent reservoir.

		Env sequences (seqs) by Q^2VOA							Env sequences (seqs) by NFL							total Env in rebound (no.)
	ID	week-2	week 12	total	clonal env seqs		overlap with NFL		week-2	week 12	total	clonal env seqs		overlap with Q^2VOA		Rebound
		no.	no.	no.	no.	(%)	no.	(%)	no.	no.	no.	no.	(%)	no.	(%)	no.
late rebounder	**9242**	28	45	73	27	37.0	10	13.7	20	25	45	11	24.4	10	22.2	19
	**9243**	25	34	59	22	37.3	7	11.9	21	12	33	4	12.1	7	21.2	13
	**9244**	38	32	70	12	17.1	6	8.6	16	15	31	9	29.0	5	16.1	31
	**9246**	6	4	10	4	40.0	4	40.0	11	11	22	8	36.4	2	9.1	20
	**9247**	3	0	3	3	100.0	3	100.0	39	42	81	79	97.5	29	35.8	35
	**9241**	45	50	95	46	48.4	44	46.3	38	20	58	7	12.1	1	17.2	22
	**9252**	41	57	98	98	100.0	98	100.0	6	6	12	10	83.3	10	83.3	14
	**9254**	18	50	68	65	95.6	65	95.6	26	62	88	84	95.5	84	95.5	NA
	**9255**	70	33	103	88	85.4	88	85.4	19	12	31	17	54.8	18	58.1	NA
early rebounder	**9245**	13			7		3	23.1	7			0	0	1	14.3	32
	**9249**	43			2		1	2.3	11			0	0	1	9.1	39
	**9253**	15			2		0	0.0	16			3	18.8	0	0.0	21
	**Total**		650							435						246
